# Evolution of Human Longevity Uncoupled from Caloric Restriction Mechanisms

**DOI:** 10.1371/journal.pone.0084117

**Published:** 2014-01-06

**Authors:** Guodong Zhao, Song Guo, Mehmet Somel, Philipp Khaitovich

**Affiliations:** 1 CAS-MPG Partner Institute for Computational Biology, Shanghai Institutes for Biological Sciences, Chinese Academy of Sciences, Shanghai, China; 2 Graduate School of Chinese Academy of Sciences, Beijing, China; 3 Max Planck Institutes for Evolutionary Anthropology, Leipzig, Germany; 4 Department of Biological Sciences, Middle East Technical University, Ankara, Turkey; University of Lausanne, Switzerland

## Abstract

Caloric restriction (CR) and chemical agents, such as resveratrol and rapamycin that partially mimic the CR effect, can delay morbidity and mortality across a broad range of species. In humans, however, the effects of CR or other life-extending agents have not yet been investigated systematically. Human maximal lifespan is already substantially greater compared to that of closely related primate species. It is therefore possible that humans have acquired genetic mutations that mimic the CR effect. Here, we tested this notion by comparing transcriptome differences between humans and other primates, with the transcriptome changes observed in mice subjected to CR. We show that the human transcriptome state, relative to other primate transcriptomes, does not match that of the CR mice or mice treated with resveratrol, but resembles the transcriptome state of *ad libitum* fed mice. At the same time, the transcriptome changes induced by CR in mice are enriched among genes showing age-related changes in primates, concentrated in specific expression patterns, and can be linked with specific functional pathways, including insulin signalling, cancer, and the immune response. These findings indicate that the evolution of human longevity was likely independent of CR-induced lifespan extension mechanisms. Consequently, application of CR or CR-mimicking agents may yet offer a promising direction for the extension of healthy human lifespan.

## Introduction

Humans stand out among primates not only with respect to cognitive abilities, bipedal locomotion, and tool production, but also with respect to maximal lifespan. During mammalian evolution, longevity has increased independently along multiple lineages, but one of the most drastic lifespan extensions can be observed in the lineage that includes humans [1],[2]. Human average lifespan in pre-modern settings was already twice as long as that of our closest living relative, the common chimpanzee [3]. Among wild chimpanzees, life expectancy at birth is ∼15 years of age, and 90% of individuals die before the age of 40 [4]. Even in captivity, chimpanzees rarely reach 50 years of age [5], [6]. By contrast, life expectancy at birth among human hunter-gatherers is 30–40 years, and 10–30% reach their 70^th^ birthday [3], [7], [8]. It has been hypothesized that the delay in human aging-related mortality evolved in parallel with a longer developmental period, which itself may have resulted from selection for an extended learning period and resource sharing abilities [4], [9], [10].

The molecular changes that caused delayed mortality and extended lifespan in humans remain unknown (reviewed in [11]). Diverse physiological processes can affect mortality and age-related functional decline, including energy and lipid metabolism, the immune response, and DNA damage repair, among others [12]–[15]. Genetic, environmental and pharmaceutical interventions directed at these and other processes have been shown to dramatically alter maximal lifespan of laboratory species by as much as 10-fold [16].

Of these interventions, one of the best-studied is caloric restriction (CR). CR, a form of dietary restriction, refers to reducing daily calorie intake by 10–40% compared to unrestricted (*ad libitum*) food intake. A key feature of the CR effect is its evolutionary conservation: CR was shown to extend maximal lifespan and/or delay morbidity in various taxa including yeast, nematode worms, fruit flies, rats, and even macaques [17]–[24]. For instance, in rodents and primates, CR has been shown to reduce the risk of several aging-associated disorders including cardiovascular disease, neurodegenerative disease, diabetes, and cancer (e.g. [18], [25]–[29]).

CR is a versatile intervention that alters the expression levels of hundreds of genes involved in a variety of biological processes, such as growth, sugar and lipid metabolism, the immune system, as well as the oxidative stress response and damage repair [19], [30]. The molecular effects induced by CR substantially overlap with the two major pathways linked with lifespan regulation in model organisms: insulin/insulin-like growth factor (IGF) signaling and target of rapamycin (TOR) signaling [31]. Pharmaceutical agents affecting these pathways and thus mimicking the CR effect can affect morbidity and lifespan in model organisms [32]. One such agent is resveratrol, a polyphenol found in grapes, that can improve health in aged mammals, including increased insulin sensitivity and cardiac function [33], [34]. Resveratrol treatment can also increase survival in mice on a high-fat diet [33], although not on a standard diet [36]. The phenotypic and molecular effects of resveratrol partially overlap with those seen in CR mice [34]. Another agent, rapamycin, inhibits TOR signaling similar to the effect of CR in model organisms [32]. In mice, treatment with rapamycin results in 9–14% life extension without any decrease in calorie intake [35].

Given the powerful influence of CR on longevity, it is possible that the evolutionary extension of human longevity involved genetic changes mimicking the physiological effect of CR, even at a well-nurtured state. Supporting this possibility, mutations affecting genes in the IGF signaling pathway, such as transcription factor *FOXO1A*, are associated with longevity among humans [36]. Further, it was reported that *FOXO1* is transcribed at higher levels in humans compared to chimpanzees [37]. Insulin and IGF levels were also reported to be higher in male chimpanzees compared to male humans [38]. If such changes did contribute to human longevity, a CR-like signature, at least in some physiological processes, should be detectable among gene expression changes separating humans from closely related shorter-lived primates: chimpanzees and macaques.

This hypothesis can be tested by comparing gene expression changes induced by CR and resveratrol in the mouse system with gene expression changes separating humans from chimpanzees and macaques, across multiple tissues and ontogenetic time intervals. In recent years, a number of studies have published mouse transcriptome data suitable for such an analysis (Table 1). One experiment used 14-month old mice subjected to 70% CR or a resveratrol diet, for 16 months [34]. Mice under both treatments showed increased health parameters relative to same-aged *ad libitum*-fed (AL) controls. Importantly, the authors reported significant correlation in gene expression changes induced by CR and resveratrol treatments, in both neocortex and heart tissue. Another study compared liver gene expression in 6 month-old mice subject to 70% CR and long-lived dwarf mice [39]. The authors found limited overlap between the two models' expression profiles, but common changes indicating increased insulin sensitivity in CR and in dwarf mice, relative to wild-type AL controls. The third study included middle-aged mice fed on a high-calorie diet (60% of calories derived from fat) for 6-months, mice on a similar diet but supplemented with resveratrol, and control mice [33]. This showed that resveratrol can improve overall health and survival under the high-calorie diet. Notably, liver gene expression changes observed in high calorie-fed mice were slowed down by resveratrol treatment. The authors also noted similarities between resveratrol-induced changes in high calorie-fed mice, and caloric restriction-induced liver expression changes observed in another experiment. Together, these datasets provide sufficient material to test the hypothesis that gene expression differences between humans and other primates could partly resemble CR-induced transcriptomic changes, such as lower insulin signaling, which, in turn, could explain the genetic basis of human longevity.

**Table 1:** Dataset information.

Dataset ID	C1	C2	C3**	S1	S2	S3	S4***
Tissues	Heart, Cerebral cortex	Liver	Liver	Liver	Liver, Heart	Cerebral cortex	Liver, Heart, Cerebral cortex
Species	Mouse	Mouse	Mouse	Human, Chimpanzee, Rhesus macaque	Human, Chimpanzee	Human, Chimpanzee, Rhesus macaque	Human, Chimpanzee, Rhesus macaque
Individuals*	5 CR, 5 AL, 5 resveratrol	8 CR, 7 AL	5 standard diet, 5 high-calorie diet, 4 resveratrol	12 Human, 12 Chimpanzee, 12 Macaque	6 Human, 5 Chimpanzee,	33 Human, 14 Chimpanzee, 34 Macaque	15 Human, 15 Chimpanzee, 4 Macaque
Platform	Affymetrix Mouse Genome 430 2.0 Array	Affymetrix Murine Genome U74A Version 2 Array	Agilent-012694 Whole Mouse Genome G4122A	Illumina Genome Analyzer II	Affymetrix Human U133plus2 arrays	Affymetrix Human Gene 1.0 ST array	Affymetrix GeneChip Human Exon 1.0 ST Arrays
Reference	[34]	[39]	[33]	[55]	[40]	[46]	GSE44147§

The datasets and samples used in the study. C1-3 refer to mouse caloric restriction/resveratrol experiments. S1-4 refer to primate gene expression experiments. CR: caloric restricted mice; AL: *ad libitum*-fed mice; resveratrol: resveratrol-treated mice.

Includes only the samples involved in our respective analysis.

This experiment compared high calorie-consuming mice with standard calorie-consuming mice.

In the S4 dataset, heart and cerebral cortex samples from rhesus macaque individuals were not present.

NCBI GEO ID for the dataset created for this study.

## Materials and Methods

### Sample collection

We collected postmortem samples of prefrontal cortex, heart and liver tissue from humans (n = 15), chimpanzees (n = 15) and rhesus macaques (n = 4) (Table 1, Table S1). All individuals were healthy adult males. The human samples were obtained from NICHD Brain and Tissue Bank for Developmental Disorders (NICHDBB) (Baltimore, MD, USA), the chimpanzee samples were obtained from the Yerkes Primate Center (Atlanta, GA, USA), the Biomedical Primate Research Center (Rijswijk, Netherlands) and from the Anthropological Institute of the University of Zurich (Swithzerland), and the macaque samples were obtained from the Suzhou Drug Safety Evaluation and Research Center (China). Frozen brain samples from male mice were provided by the animal housing center at the Max Planck Institute for Evolutionary Anthropology (Germany). All subjects had unrestricted access to food, none were obese, and none suffered from acute metabolic disorders. Mice prefrontal cortex samples were collected before mRNA isolation.

### Ethics Statement

Informed consent for the use of human tissues for research was obtained in writing from all donors or their next of kin. All non-human primates used in this study suffered sudden deaths for reasons other than their participation in this study and without any relation to the tissue used. All chimpanzees died naturally. All macaques were involved in a drug testing study as a control group and were killed by injection with a lethal dose of ketamine. All non-human primate samples were donated to the study. Biomedical Research Ethics Committee of Shanghai Institutes for Biological Sciences completed the review of the use and care of the animals in the research project (approval ID: ER-SIBS-260802P). All mice were bred, housed, and sacrificed by method of neck dislocation at the Max Planck Institute for Evolutionary Anthropology mouse facility.

### mRNA extraction, hybridization and data preprocessing

Experimental procedures of mRNA extraction followed [40], [41]. Briefly, mRNA from primate samples was hybridized to the Affymetrix GeneChip Human Exon 1.0 ST Arrays following the standard Affymetrix protocol. For data processing, we firstly mapped probes in the Human Exon 1.0 ST Chip to the human genome (hg18), chimpanzee genome (panTro2.1) and rhesus macaque genome (MMUL1.0) separately using Bowtie [42]. We then masked out probes that could not match perfectly and uniquely to any of the respective genomes. To exclude probes with signal intensities below background noise, we compared each probe's intensity to the distribution of anti-genomic probe intensities with the same GC content (the latter are designed to detect non-specific hybridization). A “detectable” probe was defined as one with intensity larger than the 95% percentile of the anti-genomic probes' intensities (comparing only to those anti-genomic probes with the same GC content). Detectable probe intensity values were log2 transformed and quantile normalized. If a transcript had more than 10 detected probes, we called this a detectable transcript. Affymetrix's Transcript Cluster Annotation file was used to map transcript clusters to Ensembl Genes (v.54). For mouse, mRNA was hybridized to Mouse Gene 1.0 ST Arrays following the same data processing procedure described above. A transcript was called detectable if it had more than 7 detected probes. All data is deposited in the NCBI Gene Expression Omnibus (GEO) database (http://www.ncbi.nlm.nih.gov/geo/) with ID GSE44147.

### Public data collection and processing

We searched the GEO database for gene expression data from caloric restriction experiments using mice, as well as gene expression data from human, chimpanzee and rhesus macaque. In total, we collected 6 datasets, which included information about three tissues: cerebral cortex, liver, and heart (Table 1). Expression profiles from each experiment were downloaded from GEO and processed following the authors' description. To facilitate comparison across species and across datasets, we used the following procedures: (1) all mouse and human gene expression datasets were annotated using mouse and human Ensembl (v.54) gene IDs, respectively, (2) mouse Ensembl gene IDs were mapped to human Ensembl gene IDs, using one-to-one human-mouse orthologous gene pairs defined by Ensembl (downloaded from the Ensembl BioMart website; http://www.biomart.org). Microarray expression data was preprocessed and summarized based on Ensembl genes, using the R [43] “affy” library [44] and in-house code.

### Effect size calculation and pair-wise comparisons of diet and species effects

**Figure 1:**
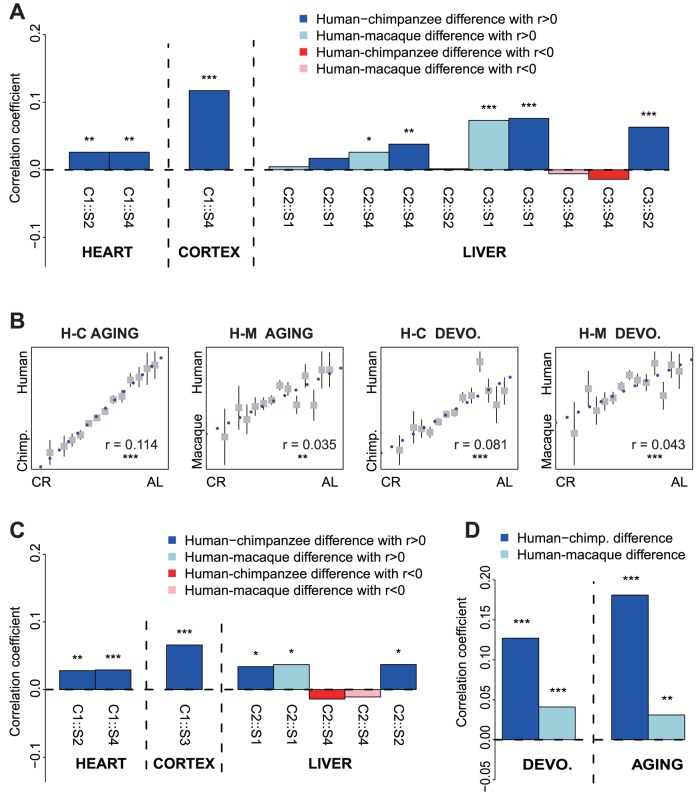
Correlations between human-specific gene expression divergence with CR-induced gene expression changes in mice. Normalized gene expression divergence (effect size) between humans and non-human primates was compared with the normalized expression difference (effect size) between CR mice and *ad libitum*-fed (AL) mice in each tissue. A: Bar plot representing the Pearson correlation coefficient between human and non-human primate differences and the CR effect in heart, cerebral cortex and liver. Each bar represents a comparison, and the text (e.g. C1::S2) below each bar gives the reference for the data sets used in a comparison (see Table 1). B: Scatter plot of primate species differences and CR effect in the cerebral cortex. The x-axis represents the effect size for human and non-human differences; the y-axis represents the effect size for CR and AL differences. Genes with similar CR-AL effect size were binned together to calculate the mean value for each group of genes and the bars represent the variance of each bin. The significance of the correlations are based on the Pearson correlation test, using all expressed genes. Pearson correlation coefficients and significance levels are shown inside the panels. H-C AGING: Correlation of human-chimpanzee differences with the CR effect for the aging period (post-adulthood). H-M AGING: Correlation of human-macaque differences with the CR effect in aging. H-C DEVO: Correlation of human-chimpanzee differences with the CR effect in postnatal development. H-M DEVO: Correlation of human-macaque differences with the CR effect in postnatal development. C: Bar plot representing the Pearson correlation coefficient between human and non-human primate differences and the resveratrol effect in cerebral cortex, heart and liver. Each bar represents a comparison, and the text (e.g. C1::S2) below each bar gives the reference for the data set used in a comparison (see Table 1). D: Bar plot representing the correlation of human and non-human primate differences with resveratrol effect in the cerebral cortex for both postnatal development and aging periods. The asterisks indicate correlation significance levels corrected for multiple testing: *: *p*<0.05; **: *p*<0.01; ***: *p*<0.001.

In order to capture the gene expression differences between two conditions (e.g. CR vs. *ad libitum* or human vs. chimpanzee), Cohen's *d* (mean difference between groups normalized by pooled standard deviation) was used as the effect size measure [45]. For each dataset, tissue, and gene, we calculated effect size for CR-*ad libitum* differences in mice, standard diet-high calorie diet differences in mice, resveratrol-control differences in mice, human-chimpanzee differences, or human-rhesus macaque differences. Next, we compared effect sizes across all detectable homologous genes between a pair of datasets (diet/resveratrol effect versus species difference effect) involving the same tissue, using the Pearson's correlation test (Figure 1). To test whether significant positive Pearson correlations (at *p*<0.05) between the diet/resveratrol effect versus the species difference effect occur more than expected by chance among the multiple datasets, we first used a binomial test. Because the comparisons are not independent, we further estimated the significance and false discovery rate (FDR) of this overall correlation using permutations. We used the following procedure: (1) we shuffled the species or treatment labels in each of the diet/resveratrol or species datasets; (2) for each shuffled dataset, we re-calculated the effect size with the same method as described above; (3) we calculated the correlations between each pair of shuffled datasets (diet/resveratrol versus species effect) involving the same tissue. This procedure was repeated 100,000 times. Finally, we calculated the significance of the number of positively correlated comparisons (*r*>0 and *p*<0.05) as the frequency among 100,000 permutations where we found at least as many positively correlated comparisons. We further calculated the FDR as the ratio between the median number of positively correlated comparisons among the 100,000 permutations, relative to the observed number of positively correlated comparisons.

### Brain age series data processing

We used an age series dataset [46] to make comparisons of transcriptome difference between humans and the other two primates, during postnatal development and in aging, separately. As subject ages were unevenly distributed across time and among species, we used interpolated points to compare expression profiles among species [47]. For interpolation, we fit spline curves with age to the expression data for each gene, and for each species, using the R function “smooth.spline” with parameters df = 3. Furthermore, in each case, to model expression changes during aging we fit regression models using chronological age, and to model changes during postnatal development we fit regression models using log-transformed age. Expression changes occur rapidly in early life and are therefore best modeled using log-transformed age, whereas expression changes occur slower after sexual maturation, and are best modeled using a linear age scale [41]. Because the three primate species have different rates of maturation and aging, we used specific age-ranges to compare their expression profiles: ages 0∼20 years for human (n = 12), 0∼12 years for chimpanzee (n = 8), and 0∼6 years for macaque (n = 16) were chosen to define the developmental period; ages 20∼98 years for human (n = 11), 12∼54 years for chimpanzee (n = 4) and 6∼27 years for macaque (n = 10) were chosen to define the aging period. The ages were chosen to correspond to ages at first reproduction and physical maturation in non-human primates [29], [48] and humans [49]. To compare between a pair of species during a specific interval, we compared the interpolated expression curves in that interval using the same effect size calculation method. We divided each specific interval (developmental period or aging period) into 10 subintervals for each species, and used the interpolated expression values at the 10 evenly distributed age points to calculate the effect size between a pair of species. Note that this procedure removes inter-individual expression variation within species, but expression variation due to age is retained. This approach further allows straightforward comparison among species with different developmental and aging rates.

### Analysis of CR-affected genes

The Welch's t-test (without assuming equal variances between groups) was employed to calculate the significance of expression differences for each gene between the CR condition and control (*ad libitum*) condition, using the R function “t.test”. Next, we performed 1000 permutations of sample labels to estimate the random significance level of expression difference. We determined the *p*-value cutoff at permutation-based false discovery rate (FDR) <10%, and all genes below this cutoff were defined as CR-affected genes. Notably, genes with higher expression level tend to have lower experimental and/or biological noise [50], leading to ascertainment bias in statistical tests for differential expression: genes with higher than average expression will have higher opportunity to pass *p*-value thresholds. This, in turn, can generate spurious overlaps when comparing significantly differentially expressed gene lists. To eliminate such possibility, we also classified genes into different groups according to their expression level and did the same analysis as above. We still found that age-related genes are significantly enriched in CR-affected genes with both high expression levels and low expression levels (data not shown). Finally, we used a non-parametric test (Mann-Whitney U (MWU), using the R “wilcox.test” function) to identify CR-affected genes, as alternative to the Welch's t-test. The results were consistent using either of the two tests. Specifically, we find 5732 CR-affected genes (at FDR <10%) using the MWU test, compared to the 5456 genes using the t-test. The two gene lists show high overlap: 5143. In addition, we confirmed that CR-affected genes identified using the MWU test are also significantly enriched in age-related genes (Fisher exact test, *p*<0.0001), are enriched in Pattern-2 genes (see below) (Fisher's exact test, Bonferroni correction, *p* = 0.012), but are not enriched in Pattern-1 or Pattern-3 genes (Fisher's exact test, Bonferroni correction, *p* = 0.09 and *p* = 1).

### Pattern identification for 3 primate species

**Figure 2:**
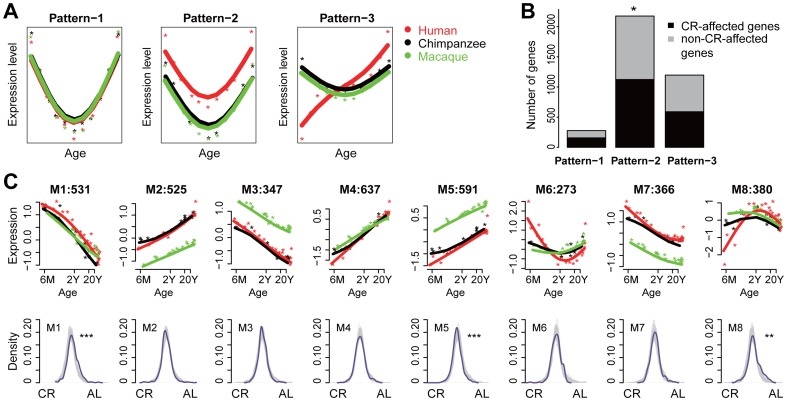
Association of the CR effect and age-related changes in brain. A: Simulated examples of expression changes with age: Pattern-1 denotes genes with expression changes with age conserved across species; Pattern-2 denotes genes with a conserved pattern of expression change with age, but also with significant average expression level differences among species; Pattern-3 denotes genes with developmental expression pattern differences among species. Points represent individuals; lines represent regression curves for each species. B: Bar plot showing the numbers of CR-affected and non-CR-affected genes among Pattern 1–3 genes. Fisher's exact test was used to test the enrichment of CR-affected genes among genes showing each of the three patterns (based on data set C1 in Table 1). C: Genes within Pattern-2 were grouped into 8 modules (M1-M8) by *k*-means clustering based on gene expression patterns across human, chimpanzee and macaque. In the upper row panels the x- and y-axes represent log2-transformed age and normalized gene expression level, respectively. Colors represent species: red: human; black: chimpanzee; green: rhesus macaque. The number of genes in each module is shown on top of each figure. In the bottom row panels the x- and y-axes represent CR-AL (*ad libitum*) effect size and the density (relative frequency) of the effect size distribution, respectively, for the same gene modules depicted in the upper row panels. The null distribution of the CR effect (generated by permuting CR labels 1000 times and recalculating effect size, shown in grey) was compared with the real distribution of CR effect size within each module (purple). The asterisks indicate the significance comparing the distribution of gene effect size within each module to the permutations. *: *p*<0.05; **: *p*<0.01; ***: *p*<0.001.

Polynomial regression models were used to identify age-related genes and non-age-related genes, as well as to test for pair-wise species differences in the human, chimpanzee and rhesus macaque age series data. For each gene, to test for significant age-related change, we used families of polynomial regression models, including linear, quadratic and/or cubic terms with individual age, using the R “lm” function, and tested significance using the F-test with the R “anova” function [43]. We further tested significant differences between age-expression trajectories between a pair of species using ANCOVA with the R “lm” and “anova” functions. We employed a method described in [46] to further group age-related genes into three patterns, represented in Figure 2A. Pattern-1: genes changing with age (at F-test p<0.01) but showing no species difference between any pair of species (at F-test p>0.1). Pattern-2: genes changing with age (at F-test p<0.01), and showing significant species differences between at least one pair of species (at F-test p<0.01) and showing no difference in developmental expression patterns among three species (at F-test p>0.1). This latter quality is tested by removing any constant expression level difference among the species (by normalizing each species' expression vector, for each gene, to the same mean), and then testing for significant differences between pairs of species. Pattern-3: genes changing with age (at F-test p<0.01), and also showing a developmental expression pattern difference among three species (at F-test p<0.01) (i.e. even after normalizing to the same mean, we find differences between the expression-age trajectories of different species).

### Clustering of expression profiles

We used the *k*-means method to cluster genes changing with age, or showing species differences, but not showing expression pattern differences among the three species (pattern-2 above), into different sets with similar expression patterns within each set. For each cluster of genes, we compared the effect size between humans and chimpanzee/rhesus macaque for these genes, with the effect size for randomly chosen genes. We then performed 1000 permutations of genes to estimate the significance of difference for each cluster of genes.

### Functional analysis

We used the DAVID Gene Set Enrichment Analysis [51] tool to test the enriched functions of each group of genes including Gene Ontology (GO) [52] and Kyoto Encyclopaedia of Genes and Genomes (KEGG) [53] pathways. Only terms with *p*-value lower than 0.05 after Bonferroni correction are reported.

### Pathway analysis

Ensembl genes within each KEGG pathway were extracted using the R CORNA package [54]. We then tested the correlation between CR effect and species effect across genes within each pathway. Only pathways with more than 5 genes were tested. We limited our analysis to human-chimpanzee differences during the aging period with or without CR, as this was the comparison with the strongest correlation between species and CR/resveratrol effects. Pathways with *p*<0.05 after Bonferroni correction for multiple testing were reported as significant.

### Expression shift analysis of mouse and human

In order to deduce how the timing of physiological changes matches between human and mouse, we used the dynamic time warping algorithm developed by Yuan et al. [47]. This can calculate the time-shift between orthologous genes' expression curves from different species, such as human and mouse. We used the above-described dataset for mouse (Table S1) containing 12 individuals between ages 2 and 904 days. Genes within the mTOR pathway, as defined in the KEGG database, were obtained using CORNA [54]; their expression profiles are shown in Table S2. For human, we used 23 human prefrontal cortex samples (aged between 0 days and 98 years) published in [46]. We used the estimated conception age of mouse and human, respectively, for the analysis in order to eliminate the prenatal development differences between mouse and human. That is, we added 19 days to mouse and 280 days to human postnatal ages, respectively. Genes with a correlation of gene expression between the human and mouse larger than 0.5 were retained for shift analysis. We used bootstrapping (sampling with replacement) to infer the mean age of human, and the relative confidence interval, corresponding to a 600 days-old mouse, both for all genes and for genes within the mTOR pathway, respectively.

## Results

### Comparison of human-specific and CR-induced expression changes in adult tissues

To investigate whether physiological differences between humans and other primates may involve “CR-like” changes in humans, we first compared CR-induced transcriptomic changes in mice, with human-specific gene expression patterns (*i.e.* expression differences between humans and chimpanzees, or human and macaques) observed in adult individuals. To do so, we used publicly available transcriptome data and additionally generated a novel transcriptome dataset from three primate tissues (Table 1). We collected the following publicly available datasets: (a) brain, heart, and liver transcriptome datasets from CR and *ad libitum*-fed mice [33], [34], [39]; (b) liver transcriptome data from mice on a standard diet and a high calorie diet [45]; (c) adult primate liver and heart transcriptome datasets [40], [55]. In addition, we generated transcriptome measurements from brain, liver and heart tissue in a total of 15 human, 15 chimpanzee and 4 rhesus macaque tissue samples using Affymetrix Exon arrays (Table 1).

Based on these data, we compared gene expression differences observed between CR and *ad libitum*-fed mice to gene expression differences between humans and the two shorter-lived primate species: chimpanzees and macaques. We found that, relative to the other two primate species, the human transcriptome was consistently closer to the transcriptome of *ad libitum*-fed mice, rather than CR mice (Figure 1A). The positive correlations between human-specific and *ad libitum*-fed transcriptome changes were observed in all comparisons conducted using brain and heart datasets, as well as all the comparisons resulting in significant correlations conducted using liver datasets (Pearson correlation test, *p*<0.05) (Figure 1A). Tested across all comparisons, this trend was highly significant (one-sided binomial test, *p*<0.0001) and could be further confirmed using sample permutation analysis (*p* = 0.0002). These results do not support the notion that human longevity has been driven by “CR-like” genetic changes affecting the human transcriptome. Rather, the human transcriptome corresponds to a “well-fed” mouse phenotype, possibly reflecting augmented dietary caloric content during human evolution, relative to the diets of non-human primate species [3], .

### Comparison of human-specific expression changes across lifetime and CR-induced expression changes

In mammals, a large number of genes change their expression levels during lifetime, from birth until old age [41]. Since the above-described analysis only involved adults, we additionally tested the similarity between the CR state and human-specific expression changes using published prefrontal cortex time-series data, where gene expression was measured in 23 human, 12 chimpanzee and 25 rhesus macaque individuals of different age [46]. Using these data, we compared gene expression differences induced by CR with gene expression differences between humans and shorter-lived primate species, chimpanzees and macaques, during human postnatal development (0–20 years of human age) and aging (20–98 years of human age). Indeed, we again found that during both ontogenetic periods the human brain transcriptome, relative to the chimpanzee and rhesus macaque transcriptomes, showed significantly greater similarity to the transcriptome of *ad libitum*-fed mice than to that of CR mice (Pearson correlation, *p*<0.01) (Figure 1B). Notably, among all comparisons, AL *versus* CR differences showed the strongest correlation with human *versus* chimpanzee differences during aging. These results further imply that the lower mortality and higher longevity characteristic of humans is not caused by the “CR-like” state of the human transcriptome at any stage of postnatal ontogenesis.

### Comparison of human-specific and resveratrol-induced expression changes

Resveratrol, a natural phenol, has been shown to successfully increase the survival of mice fed high-calorie diets [33] and to extend lifespan in several species [33], [58], [59]. In mice, transcriptional changes under resveratrol have also been reported to mimic the changes mediated by caloric restriction [60]. We thus asked how the transcriptional effects of a lifespan-extending drug like resveratrol may compare with gene expression differences observed among primate species. To do so, we used results from two experiments that studied the expression changes induced by resveratrol in the mouse brain, liver and heart [33], [34]. Similar to the comparison between CR and *ad libitum*-fed mice, we found that the human transcriptome, when compared to the transcriptomes of non-human primates, was more similar to the untreated mice transcriptome than to the resveratrol-treated mice transcriptome. This effect was consistent across tissues and observed for all comparisons conducted in the brain and heart, as well as for three out of the five comparisons conducted in the liver (Figure 1C). The trend was highly significant across data sets (one-sided binomial test *p*<0.00001, permutation test *p* = 0.0001). We also found significant correlations in the same direction when comparing the resveratrol effect to human *versus* non-human primate differences during postnatal development or aging (Figure 1D). No comparison showed a significant trend in the opposite direction. This result demonstrates that transcriptome features separating humans from shorter-lived primates also do not mimic the changes induced by resveratrol treatment.

### Functional association between CR effect and age-related changes in primates

Caloric restriction has been shown to retard the aging process and partially attenuate age-related gene expression changes [61]–[63]. In agreement with this observation, we found significant over-representation of CR-affected genes identified in the mice brain, among genes undergoing age-related expression changes in the human prefrontal cortex [41] (Fisher's exact test, *p*<0.001) (Materials and Methods). This association was also valid after controlling for gene expression level as a potential confounding factor (Materials and Methods). We further classified age-related genes found in the human brain into three categories based on the conservation of their expression profiles among humans, chimpanzees and rhesus macaques, and following the methodology suggested elsewhere [46] (Materials and Methods): (Pattern-1) genes with a developmental expression pattern and conserved expression level among the three species; (Pattern-2) genes with a conserved developmental expression pattern, but expressed at different levels in the three species; and (Pattern-3) genes showing a developmental pattern difference among the species (Figure 2A). We found that CR-affected genes are enriched in Pattern-2 genes (Fisher's exact test, Bonferroni correction, *p* = 0.019), but not in Pattern-1 or Pattern-3 genes (Fisher's exact test, Bonferroni correction, *p* = 0.06 and *p* = 1) (Figure 2B).

We next focused on Pattern-2 genes to search for specific age-related expression modules, and their corresponding functional processes, that overlap with transcriptome changes found in CR. Using the *k*-means clustering algorithm, we classified Pattern-2 genes into eight modules based on their prefrontal cortex expression profiles across the three species (Figure 2C). Three out of these eight modules, module 1, 5 and 8, showed a significant correlation between age-related changes found in primates and expression changes induced by CR (Figure 2C). In agreement with the general analysis of transcriptome changes described above, in all three modules the human expression state could be associated with *ad libitum*-fed mice, and the chimpanzee and macaque expression state - with CR mice (Figure 2C).

We then investigated the functional properties of module 1, 5 and 8 genes using Gene Ontology (GO) terms and KEGG pathways. Module 1 genes were enriched for genes involved in glycosylation, known to regulate nutrient-mediated signalling [64]. Module 5 genes were enriched in the GO term “mitochondrion”, the site of oxidative metabolism in eukaryotes, which is closely linked with aging and CR response [23], [65], [66]. Module 8 genes were enriched in GO terms associated with the plasma membrane and synapses – functions also shown to be affected by CR [67] (Table S3). These results indicate that several pathways associated with CR effects show the same trajectory of age-related changes in humans, chimpanzees and macaques, but display expression level shift in humans in the direction of the *ad libitum*-fed expression state.

To further investigate the association between the CR-induced and the human-specific transcriptome changes at the pathway level, we tested the correlation in the expression differences between humans and chimpanzees during brain aging, and the CR-induced expression changes in the mouse brain, for each of the KEGG pathways. Notably, the human-chimpanzee comparison during brain aging (relative to comparisons in other tissues, human-macaque comparisons, and species differences during postnatal development) had shown the strongest overall correlation between the CR effect and species differences (Figure 1A–B).

Of the 230 KEGG pathways tested, genes in 22 pathways showed significant positive correlations, indicating similarity between the human transcriptome and the expression state of *ad-libitum* fed mice (Pearson correlation, *p*<0.05). Notably, these 22 pathways included the insulin signaling, as well as a number of other functional processes involved in development, cell growth, cancer, and immunity (Table S4). This is especially interesting given that insulin signaling is one of the two major pathways involved in lifespan regulation in multiple species, including humans [68], [69]. Meanwhile, improved immune function and decreased incidence of cancer are hallmarks of CR in both mice and primates [14], [18]. Notably, no pathway showed negative correlations between human *versus* chimpanzee differences and AL *versus* CR differences.

### Association between the effect of rapamycin and age-related changes in primates

The results above suggest that during human evolution, due to either environmental or genetic effects, human physiology moved farther away from the physiological states induced by CR- or resveratrol treatment. This implies that CR or treatments with CR-mimetics in humans may yet have the potential to induce transcriptome changes associated with greater longevity. One such particular treatment involves the suppression of the mammalian target of rapamycin protein (mTOR) pathway. mTOR signaling regulates cell growth and cell mortality in response to nutrient intake [70], and its suppression has been implicated to play a central role in the CR response [24], [71]. Rapamycin, a bacterial product that interacts directly with the mTOR gene, can increase lifespan in mice, even when administered at an advanced age, such as 600 days [35].

**Figure 3:**
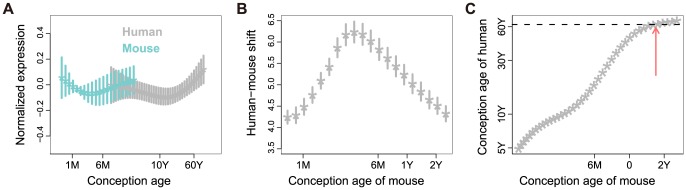
Time-shift between human and mouse expression profiles in the mTOR pathway. A: mTOR pathway gene expression curves of human and mouse prefrontal cortex. The x-axis shows log-transformed age from conception (“conception age”) values for both human and mouse, and the y-axis shows the normalized gene expression value. Each asterisk represents the mean expression value for a certain age point and horizontal bars represent the expression variance of genes within the mTOR pathway (only 42 of the 52 genes that showed substantial correlation (Pearson r>0.5) with the average pathway profile were included). B: Human-mouse time-shift. The x-axis shows the age from conception of mouse, and the y-axis shows the time-shift of human age relative to mouse age. Each asterisk represents the average time shift of genes within the mTOR pathway, and the bars show the variance of each time point. C: Scatter plot of human age plotted against mouse age according to the human-mouse time-shift values. The x- and y-axes show the age from conception of mouse and human, respectively. The red arrow indicates the point where human age equals 600 days of mouse age based on the time-shift calculation for the mTOR pathway genes.

The discovery of the life-extending rapamycin effect in mice has raised the possibility that rapamycin-like therapies administered in middle-aged humans could also lead to the extension of healthy lifespan. At what age could such a therapy be successfully administered in humans? To answer this question, we determined the physiological age of the human brain that corresponds to the physiological state of 600 day-old mice, particularly with respect to gene expression changes in mTOR pathway genes. Specifically, we calculated the time-shift between human and mouse gene expression time-series data for all expressed genes, and for genes in the mTOR pathway, using an optimized dynamic time-warping algorithm [47]. The transcriptome-wide alignment of human and mouse expression time-series showed that the brain transcriptomic state of 600 day-old mice corresponds to the brain transcriptomic state of a 66-year old human +/−1 year (95% bootstrap confidence interval) (Figure S1). For mTOR pathway genes, however, the corresponding human age was on average slightly younger: 62 years +/−10 years (95% bootstrap confidence interval) (Figure 3). Assuming that transcriptome changes in the brain reflect the rate of overall senescence, this result indicates that rapamycin-like therapies have a potential to be successfully introduced in humans at least until 62 years of age.

## Discussion

Multiple lines of evidence indicate that CR and CR-mimicking drugs and mutations can improve health and/or increase maximal lifespan in a vast variety of species, including mice and macaques [18], [21], [22]. At the same time, the maximal lifespan of humans is already substantially longer than the maximal lifespan of other primate species, including closely related ones. This has raised the question whether mutations that shifted human physiology toward a “CR-like” state may have contributed to lifespan extension during human evolution.

Here we show that human-specific transcriptome changes are more similar to the transcriptome of *ad libitum*-fed mice rather than CR mice. This result could be replicated in multiple tissues, and multiple datasets, generated by a number of independent research groups and observed at different stages of human postnatal lifespan.

There remains the possibility that some of the mutations involved in the evolution of human longevity have effects resembling that of CR, such as improved stress or immune responses. Nevertheless, because CR can induce massive gene expression changes in multiple tissues [17], [30], we would expect any substantial contribution of a CR-like physiological change to human longevity to be detectable in at least some of the human tissues, or some physiological processes, at the transcriptome level. Instead, we observe a substantial shift in the human transcriptome towards *ad libitum*-fed mice across all the tissues and functional pathways examined, including insulin signaling, cancer and immune pathways. Therefore, our observations do not support the hypothesis postulating that mutations inducing a general CR-like physiological state, even in the presence of ample nutrition, contributed to the extended longevity of the human species. Our results instead may reflect a physiological shift in human tissues induced by a greater calorie and nutritional content in the average human diet, compared to the fruit and vegetable-based diets of chimpanzees and macaques [3], [56], [57], [72].

These findings also relate to the discussion around whether CR, or CR-mimicking drugs, such as rapamycin, could significantly lower morbidity and extend lifespan in humans [73]–[75]. It has recently been demonstrated that CR and CR-like effects can significantly lower mortality in primates by induction of a physiological state that protects against age-related disease in various tissues, including liver, heart, and brain [19], [21], at least under certain CR and genetic background conditions [76]. Accordingly, lower calorie intake has been suggested to improve health and extend lifespan in humans. For example, a six-year-long application of CR to a set of volunteers was reported to cause physiological changes that mirror those observed in CR mice, including lower blood cholesterol and insulin and a reduced risk of atherosclerosis [77]. Even six months of CR treatment could improve cardiovascular health in non-obese humans [78]. Another line of evidence in the same direction is the average longer lifespan of Okinawans (1.5 years longer than the Japanese average); this has been attributed to their lower caloric intake (83% of Japanese average in the 1970's), in addition to other genetic factors [79]. Finally, mutations in the *FOXO1A* gene, a key transcription factor implicated in the CR effect in model species, are among the few loci associated with variation in human longevity [36], further implying possible beneficial effects of CR on human health. However, various researchers have remained skeptical on the possible effectiveness of CR in extending modern human lifespan [73]. For example, Shanley and Kirkwood have argued that the CR response among animals does not have a common origin, and we should not expect a human response similar to that in model organisms [80]. Phelan and Rose have pointed out that large mammals may not respond to energy restriction as intensely as small ones [81]. This is partly because small mammals allocate a larger proportion of their resources to reproduction, and under dietary restriction, this can be efficiently reallocated towards maintenance and longevity. Accordingly, lifespan extensions seen in mice may not be observed in large mammals like humans. Meanwhile, Le Bourg has argued that CR-like responses to limited food availability would be advantageous for small mammal lineages, because migrating long distances is not an option for small species [82]. However, large mammals can migrate in times of famine, and therefore CR responses would not be selected for.

Our results indicate that humans do not show a transcriptome state compatible with the CR effect. On the contrary, human physiology has moved further away from a state shaped by modest calorie intake that we observe in other primates, including macaques. As CR can result in significant retardation of aging-related changes in macaques [21], [76], our findings suggest that CR or CR-mimicking drugs could still have the potential to affect human physiology toward a substantial extension of healthy lifespan, and improvements in humans could be more pronounced even than those seen in macaques.

## Supporting Information

Figure S1 **The transcriptome-wide alignment of human and mouse expression time-series.** A: Human-mouse time-shift curve based on the transcriptome-wide alignment of human and mouse expression time-series. The x-axis shows the age from conception of mouse, and the y-axis shows the time-shift of human age relative to mouse age. Each asterisk represents the average time-shift of genes expressed in human and macaque time-series data. B: Scatter plot of human age *versus* mouse age according to the human-mouse time shift. The x- and y-axes show the conception age of mouse and human, respectively. The red arrow indicates the point where age in humans equals to 600 days in mouse based on the time-shift calculation for all genes expressed in human and mouse times series data.
(EPS)

Table S1 Sample information.
(DOCX)

Table S2 Expression profile of genes within the mTOR pathway across mouse age-series samples.
(DOCX)

Table S3 Functional categories enriched in Module 8 genes.
(DOCX)

Table S4 Correlation between (a) human vs. chimpanzee expression differences during brain aging, and (b) ad libitum vs. CR expression differences in the mouse brain, for genes in each of 230 KEGG pathways.
(DOCX)
