# Supplementary material for: Evolution of Human Longevity Uncoupled from Caloric Restriction Mechanisms
Source: PLoS One. 2014 Jan 6;9(1):e84117. doi: 10.1371/journal.pone.0084117 (PMC3882206; doi:10.1371/journal.pone.0084117)
Supplement: Table S1 — Sample information. (DOCX) [file pone.0084117.s002.docx]

**Table S1:** Sample information

| **Sample_ID** | **Tissue** | **Species** | **Age*** |
| --- | --- | --- | --- |
| CB1Ex | prefontal cortex | *Pan troglodytes* | adult |
| CB2Ex | prefontal cortex | *Pan troglodytes* | adult |
| CB3Ex | prefontal cortex | *Pan troglodytes* | adult |
| CB4Ex | prefontal cortex | *Pan troglodytes* | adult |
| CB5Ex | prefontal cortex | *Pan troglodytes* | adult |
| HB1Ex | prefontal cortex | *Homo sapiens* | adult |
| HB2Ex | prefontal cortex | *Homo sapiens* | adult |
| HB3Ex | prefontal cortex | *Homo sapiens* | adult |
| HB4Ex | prefontal cortex | *Homo sapiens* | adult |
| HB5Ex | prefontal cortex | *Homo sapiens* | adult |
| CH1Ex | heart | *Pan troglodytes* | adult |
| CH2Ex | heart | *Pan troglodytes* | adult |
| CH3Ex | heart | *Pan troglodytes* | adult |
| CH4Ex | heart | *Pan troglodytes* | adult |
| CH5Ex | heart | *Pan troglodytes* | adult |
| HH1Ex | heart | *Homo sapiens* | adult |
| HH2Ex | heart | *Homo sapiens* | adult |
| HH3Ex | heart | *Homo sapiens* | adult |
| HH4Ex | heart | *Homo sapiens* | adult |
| HH5Ex | heart | *Homo sapiens* | adult |
| C1l | liver | *Pan troglodytes* | adult |
| C3l** | liver | *Pan troglodytes* | adult |
| C4l | liver | *Pan troglodytes* | adult |
| C5l | liver | *Pan troglodytes* | adult |
| C6l | liver | *Pan troglodytes* | adult |
| C7l | liver | *Pan troglodytes* | adult |
| H2l | liver | *Homo sapiens* | adult |
| H3l | liver | *Homo sapiens* | adult |
| H4l | liver | *Homo sapiens* | adult |
| H5l | liver | *Homo sapiens* | adult |
| H6l | liver | *Homo sapiens* | adult |
| H7l** | liver | *Homo sapiens* | adult |
| R1l | liver | *Macaca mulatta* | adult |
| R2l | liver | *Macaca mulatta* | adult |
| R3l | liver | *Macaca mulatta* | adult |
| R5l | liver | *Macaca mulatta* | adult |
| mouse_brain_cortex_1 | prefontal cortex | *Mus musculus* | 2 |
| mouse_brain_cortex_2 | prefontal cortex | *Mus musculus* | 2 |
| mouse_brain_cortex_3 | prefontal cortex | *Mus musculus* | 5 |
| mouse_brain_cortex_4 | prefontal cortex | *Mus musculus* | 11 |
| mouse_brain_cortex_5 | prefontal cortex | *Mus musculus* | 20 |
| mouse_brain_cortex_6 | prefontal cortex | *Mus musculus* | 32 |
| mouse_brain_cortex_7 | prefontal cortex | *Mus musculus* | 61 |
| mouse_brain_cortex_8 | prefontal cortex | *Mus musculus* | 122 |
| mouse_brain_cortex_9 | prefontal cortex | *Mus musculus* | 184 |
| mouse_brain_cortex_10 | prefontal cortex | *Mus musculus* | 365 |
| mouse_brain_cortex_11 | prefontal cortex | *Mus musculus* | 649 |
| mouse_brain_cortex_12 | prefontal cortex | *Mus musculus* | 904 |

* Age of mice in months

** These samples were excluded from the analysis because they appeared as outliers in PCA and cluster analyses
