# Supplementary material for: Evolution of Human Longevity Uncoupled from Caloric Restriction Mechanisms
Source: PLoS One. 2014 Jan 6;9(1):e84117. doi: 10.1371/journal.pone.0084117 (PMC3882206; doi:10.1371/journal.pone.0084117)
Supplement: Table S2 — Expression profile of genes within the mTOR pathway across mouse age-series samples. (DOCX) [file pone.0084117.s003.docx]

**Table S2:** Expression profile of genes within the mTOR pathway across mouse age-series samples

| **Ensembl Gene ID** | **mouse brain cortex 1** | **mouse brain cortex 2** | **mouse brain cortex 3** | **mouse brain cortex 4** | **mouse brain cortex 5** | **mouse brain cortex 6** | **mouse brain cortex 7** | **mouse brain cortex 8** | **mouse brain cortex 9** | **mouse brain cortex 10** | **mouse brain cortex 11** | **mouse brain cortex 12** |
| --- | --- | --- | --- | --- | --- | --- | --- | --- | --- | --- | --- | --- |
| ENSMUSG00000026254 | 1.646 | 1.156 | 1.886 | -0.642 | -0.184 | -0.923 | 0.037 | -0.896 | -0.508 | -0.322 | -0.354 | -0.897 |
| ENSMUSG00000019699 | 2.111 | 0.828 | 1.174 | 0.242 | -1.552 | -1.01 | -0.633 | -0.013 | -0.581 | -0.176 | 0.075 | -0.465 |
| ENSMUSG00000003068 | 1.204 | 0.532 | 1.958 | -1.431 | -0.987 | -0.509 | -0.738 | -0.371 | -0.58 | 0.777 | 0.582 | -0.437 |
| ENSMUSG00000020053 | -0.399 | 2.375 | 1.187 | 1.023 | -0.02 | -0.392 | -0.443 | -0.556 | -0.475 | -0.871 | -0.914 | -0.514 |
| ENSMUSG00000020108 | 0.211 | 0.406 | 1.887 | 0.111 | 1.562 | -0.865 | -1.368 | -1.388 | -0.239 | -0.408 | 0.071 | 0.019 |
| ENSMUSG00000025583 | -1.049 | -1.456 | -0.817 | -1.601 | 0.319 | -0.084 | 0.999 | 1.091 | 0.109 | 1.166 | 0.712 | 0.612 |
| ENSMUSG00000004798 | 1.743 | 1.095 | 1.475 | 0.821 | -0.075 | -0.42 | -0.7 | -0.574 | -0.739 | -1.053 | -0.892 | -0.68 |
| ENSMUSG00000020516 | 2.297 | 1.189 | 1.114 | -0.029 | -0.284 | -0.581 | -0.683 | -0.461 | -0.706 | -0.155 | -0.901 | -0.8 |
| ENSMUSG00000069631 | -1.153 | -0.41 | -0.77 | -0.181 | -1.417 | 1.058 | 0.012 | 0.187 | 0.894 | -0.437 | 0.043 | 2.173 |
| ENSMUSG00000021109 | 1.428 | 1.475 | 1.368 | 1.035 | -0.392 | -0.391 | -0.823 | -0.791 | -0.674 | -0.919 | -0.835 | -0.481 |
| ENSMUSG00000020573 | -0.759 | -1.204 | -0.843 | 0.731 | -0.869 | 0.468 | 0.515 | -0.147 | -0.191 | -0.195 | -0.034 | 2.528 |
| ENSMUSG00000001729 | 1.886 | 1.464 | 1.479 | -0.215 | -0.224 | -0.624 | -1.014 | -0.724 | -0.396 | -0.434 | -0.544 | -0.656 |
| ENSMUSG00000041417 | 1.336 | 1.584 | 1.6 | 0.362 | -0.003 | -0.864 | -0.61 | -0.653 | -0.289 | -1.225 | -0.376 | -0.863 |
| ENSMUSG00000021981 | -0.961 | -1.856 | -1.256 | 0.04 | -0.977 | 0.087 | 0.747 | 0.791 | 0.75 | 0.787 | 0.852 | 0.997 |
| ENSMUSG00000050697 | 0.265 | 1.365 | 1.018 | -0.774 | -1.833 | -0.907 | -0.097 | 0.428 | 0.997 | -0.889 | -0.556 | 0.982 |
| ENSMUSG00000050310 | 0.203 | -0.172 | -0.773 | -0.995 | 0.828 | -1.865 | -0.643 | 1.31 | 0.469 | 1.668 | 0.128 | -0.159 |
| ENSMUSG00000058655 | 0.779 | 1.247 | 1.901 | -0.116 | 0.094 | -1.229 | -0.953 | -1.622 | -0.292 | 0.003 | -0.062 | 0.252 |
| ENSMUSG00000063358 | -1.97 | -1.685 | -0.96 | 0.392 | 1.334 | 0.336 | 0.581 | 0.422 | 0.436 | 0.706 | 0.313 | 0.094 |
| ENSMUSG00000023809 | -2.304 | -1.604 | -0.545 | 0.286 | 0.602 | 0.219 | 0.903 | 0.546 | 0.32 | 0.82 | 0.657 | 0.1 |
| ENSMUSG00000024122 | -1.875 | -1.585 | -1.199 | -0.362 | 0.429 | 0.471 | 0.802 | 0.849 | 0.627 | 0.859 | 0.589 | 0.395 |
| ENSMUSG00000024142 | 1.965 | 0.809 | 1.104 | -0.678 | -0.276 | -0.87 | 1.28 | -0.584 | -0.928 | -0.483 | -0.684 | -0.654 |
| ENSMUSG00000002496 | -0.414 | -0.315 | 0.823 | -2.815 | 0.537 | -0.112 | 0.466 | -0.088 | 1.068 | 0.623 | 0.266 | -0.04 |
| ENSMUSG00000023951 | -1.57 | -1.105 | -0.231 | -0.933 | 2.052 | 0.371 | -0.052 | -0.037 | 0.102 | 1.302 | -0.353 | 0.455 |
| ENSMUSG00000024830 | -1.16 | -0.526 | -1.591 | -0.481 | 0.674 | -0.248 | 2.196 | -0.125 | -0.108 | 0.9 | -0.171 | 0.64 |
| ENSMUSG00000024962 | -0.591 | -1.476 | -1.543 | -0.394 | -0.366 | 0.583 | 0.671 | -0.377 | -0.199 | 0.972 | 1.051 | 1.667 |
| ENSMUSG00000026812 | 1.655 | 0.495 | 0.495 | -1.762 | 0.79 | -1.799 | -0.008 | 0.489 | -0.297 | -0.393 | 0.533 | -0.2 |
| ENSMUSG00000027665 | 1.873 | 1.669 | 0.59 | -1.458 | -0.352 | -0.098 | 0.035 | 0.308 | -0.983 | -0.209 | -0.535 | -0.841 |
| ENSMUSG00000028698 | -0.351 | 1.58 | 0.508 | 1.435 | 0.984 | -0.056 | -0.072 | -0.31 | 0.066 | -1.095 | -1.053 | -1.636 |
| ENSMUSG00000028991 | -1.903 | -2.046 | 0.086 | 0.199 | 0.564 | 0.249 | 0.229 | 0.409 | -0.125 | 1.452 | 0.562 | 0.326 |
| ENSMUSG00000028518 | 1.676 | 1.078 | 0.998 | 0.759 | -0.185 | -0.693 | -1.066 | -1.098 | 0.535 | -1.191 | 0.126 | -0.939 |
| ENSMUSG00000003644 | 1.781 | 0.341 | -0.13 | -0.226 | -0.22 | -1.101 | -0.465 | -1.833 | 1.227 | -0.494 | 0.095 | 1.024 |
| ENSMUSG00000039936 | 0.826 | 1.665 | 2.087 | -0.052 | -0.446 | -0.758 | -0.446 | -1.007 | -0.344 | -0.361 | -0.952 | -0.212 |
| ENSMUSG00000028945 | -0.448 | -0.195 | -1.26 | 1.525 | 0.328 | 1.63 | -0.382 | 0.435 | -1.78 | 0.451 | 0.253 | -0.557 |
| ENSMUSG00000029512 | 2.125 | 1.535 | 0.935 | -0.19 | -0.895 | 0.034 | -0.482 | -0.925 | -0.415 | -0.319 | -0.519 | -0.884 |
| ENSMUSG00000002413 | -1.534 | -1.775 | -0.793 | -0.536 | 1.048 | -0.488 | 0.36 | 0.926 | 0.367 | 1.047 | 0.882 | 0.496 |
| ENSMUSG00000063065 | -0.774 | 0.072 | 1.247 | 1.948 | 0.921 | -1.243 | -1.219 | -0.618 | -0.356 | -0.229 | 0.662 | -0.411 |
| ENSMUSG00000031490 | -0.061 | -0.24 | -0.244 | 0.201 | -2.253 | 1.166 | -1.119 | -0.131 | -0.061 | 0.907 | 0.358 | 1.476 |
| ENSMUSG00000031520 | 1.678 | 1.522 | 1.611 | -0.363 | -0.084 | -0.468 | -0.942 | -0.907 | -0.408 | -0.38 | -0.853 | -0.405 |
| ENSMUSG00000031834 | 2.27 | -0.201 | 0.906 | -0.206 | 0.198 | 0.561 | -0.883 | -1.357 | -1.202 | 0.37 | 0.146 | -0.602 |
| ENSMUSG00000032308 | -0.971 | -1.882 | -0.658 | -0.778 | 0.876 | 0.213 | 1.452 | -0.422 | 0.217 | 0.94 | -0.177 | 1.19 |
| ENSMUSG00000032462 | -0.996 | -1.227 | 0.346 | -1.066 | -0.563 | 2.194 | 0.843 | 0.988 | -0.284 | 0.389 | -0.306 | -0.318 |
| ENSMUSG00000031309 | -0.651 | -1.987 | -1.48 | -0.33 | 0.836 | 0.977 | 0.848 | 0.634 | 1.186 | 0.251 | -0.291 | 0.007 |
| ENSMUSG00000031380 | 2.027 | -1.076 | 0.34 | 0.025 | 0.023 | -0.508 | -0.768 | -1.749 | 0.796 | -0.439 | 0.685 | 0.644 |
| ENSMUSG00000025665 | 1.772 | 1.445 | 1.508 | 0.308 | -0.484 | -0.484 | -0.516 | -0.9 | -0.652 | -0.521 | -0.933 | -0.543 |
