# Supplementary material for: Evolution of Human Longevity Uncoupled from Caloric Restriction Mechanisms
Source: PLoS One. 2014 Jan 6;9(1):e84117. doi: 10.1371/journal.pone.0084117 (PMC3882206; doi:10.1371/journal.pone.0084117)
Supplement: Table S3 — Functional categories enriched in Module 8 genes. (DOCX) [file pone.0084117.s004.docx]

**Table S3:** Functional categories enriched in Module 8 genes, identified using the DAVID Gene Set Enrichment Analysis tool (Materials and Methods). Category: original database; Term: enriched terms; Count: Genes involved in the term; %: percentage of genes involved in the term; P-value: Fisher Exact P-value; Genes: genes involved in the term; FDR: false discovery rate.

| **Category** | **Term** | **Count** | **%** | **P-value** | **Genes** | **Fold Enrichment** | **Bonferroni corrected P-value** | **FDR** |
| --- | --- | --- | --- | --- | --- | --- | --- | --- |
| UP SEQ FEATURE | Topological domain: Extracellular | 97 | 18.27 | 2.92E-07 | ENSG00000173621, ENSG00000138759, ENSG00000167178, ENSG00000100678, ENSG00000139636, ENSG00000150086, ENSG00000110076, ENSG00000074855, ENSG00000148680, ENSG00000113248, ENSG00000130827, ENSG00000218336, ENSG00000196338, ENSG00000198910, ENSG00000134313, | 1.62 | 4.57E-04 | 4.87E-04 |
| UP SEQ FEATURE | Glycosylation site: N-linked (GlcNAc...) | 141 | 26.55 | 2.11E-06 | ENSG00000173621, ENSG00000138759, ENSG00000167178, ENSG00000100678, ENSG00000151079, ENSG00000150086, ENSG00000187498, ENSG00000110076, ENSG00000148680, ENSG00000113248, ENSG00000130827, ENSG00000218336, ENSG00000196338, ENSG00000198910, ENSG00000165996, | 1.41 | 3.30E-03 | 3.53E-03 |
| SP PIR KEYWORDS | Glycoprotein | 145 | 27.31 | 9.35E-06 | ENSG00000173621, ENSG00000138759, ENSG00000167178, ENSG00000100678, ENSG00000151079, ENSG00000150086, ENSG00000187498, ENSG00000110076, ENSG00000148680, ENSG00000113248, ENSG00000130827, ENSG00000218336, ENSG00000196338, ENSG00000198910, ENSG00000165996, | 1.37 | 3.45E-03 | 1.29E-02 |
| GOTERM CC FAT | GO:0031224~intrinsic to membrane | 185 | 34.84 | 1.25E-05 | ENSG00000173621, ENSG00000138759, ENSG00000125744, ENSG00000167178, ENSG00000181830, ENSG00000117598, ENSG00000139636, ENSG00000100678, ENSG00000151079, ENSG00000150086, ENSG00000110076, ENSG00000074855, ENSG00000148680, ENSG00000133935, ENSG00000113248, | 1.26 | 4.11E-03 | 1.70E-02 |
| UP SEQ FEATURE | Topological domain: Cytoplasmic | 127 | 23.92 | 3.33E-06 | ENSG00000173621, ENSG00000138759, ENSG00000167178, ENSG00000100678, ENSG00000139636, ENSG00000150086, ENSG00000110076, ENSG00000074855, ENSG00000148680, ENSG00000113248, ENSG00000130827, ENSG00000218336, ENSG00000196338, ENSG00000198910, ENSG00000153395, | 1.43 | 5.21E-03 | 5.57E-03 |
| SP PIR KEYWORDS | Neurogenesis | 20 | 3.77 | 2.37E-05 | ENSG00000169855, ENSG00000073584, ENSG00000136261, ENSG00000167178, ENSG00000080503, ENSG00000117713, ENSG00000176887, ENSG00000113758, ENSG00000126603, ENSG00000160360, ENSG00000134072, ENSG00000106852, ENSG00000186487, ENSG00000153266, ENSG00000082014, | 2.80 | 8.72E-03 | 3.26E-02 |
| SP PIR KEYWORDS | Immunoglobulin domain | 25 | 4.71 | 4.02E-05 | ENSG00000145681, ENSG00000173621, ENSG00000167178, ENSG00000154639, ENSG00000169783, ENSG00000166257, ENSG00000113805, ENSG00000166250, ENSG00000173114, ENSG00000170017, ENSG00000105426, ENSG00000196083, ENSG00000198910, ENSG00000154721, ENSG00000169855, | 2.38 | 1.48E-02 | 5.54E-02 |
| SP PIR KEYWORDS | Cell adhesion | 27 | 5.08 | 6.48E-05 | ENSG00000113361, ENSG00000152092, ENSG00000140945, ENSG00000154639, ENSG00000169760, ENSG00000197991, ENSG00000110076, ENSG00000198561, ENSG00000113805, ENSG00000113248, ENSG00000137801, ENSG00000196338, ENSG00000170017, ENSG00000105426, ENSG00000198910, | 2.23 | 2.37E-02 | 8.94E-02 |
| SP PIR KEYWORDS | Transmembrane | 170 | 32.02 | 7.30E-05 | ENSG00000173621, ENSG00000138759, ENSG00000125744, ENSG00000167178, ENSG00000181830, ENSG00000117598, ENSG00000139636, ENSG00000100678, ENSG00000151079, ENSG00000150086, ENSG00000110076, ENSG00000074855, ENSG00000148680, ENSG00000133935, ENSG00000113248, | 1.28 | 2.66E-02 | 1.01E-01 |
