# Supplementary material for: Evolution of Human Longevity Uncoupled from Caloric Restriction Mechanisms
Source: PLoS One. 2014 Jan 6;9(1):e84117. doi: 10.1371/journal.pone.0084117 (PMC3882206; doi:10.1371/journal.pone.0084117)
Supplement: Table S4 — Correlation between (a) human vs. chimpanzee expression differences during brain aging, and (b) ad libitum vs. CR expression differences in the mouse brain, for genes in each of 230 KEGG pathways. (DOCX) [file pone.0084117.s005.docx]

**Table S4:** Correlation between (a) human *vs.* chimpanzee expression differences during brain aging, and (b) *ad libitum vs*. CR expression differences in the mouse brain, for genes in each of 230 KEGG pathways.

| **KEGG term** | **Correlation coefficient** | **P-value** | **Number of genes** |
| --- | --- | --- | --- |
| Chronic_myeloid_leukemia | 0.44 | 6.31E-04 | 56 |
| T_cell_receptor_signaling_pathway | 0.37 | 2.58E-03 | 63 |
| Notch_signaling_pathway | 0.51 | 4.73E-03 | 29 |
| Metabolic_pathways | 0.11 | 6.96E-03 | 648 |
| Glioma | 0.35 | 9.86E-03 | 55 |
| Pathways_in_cancer | 0.18 | 1.14E-02 | 203 |
| Prostate_cancer | 0.31 | 1.21E-02 | 66 |
| Fc_epsilon_RI_signaling_pathway | 0.38 | 1.30E-02 | 43 |
| B_cell_receptor_signaling_pathway | 0.36 | 1.36E-02 | 46 |
| Calcium_signaling_pathway | 0.25 | 1.36E-02 | 96 |
| MAPK_signaling_pathway | 0.19 | 1.37E-02 | 171 |
| Melanoma | 0.35 | 1.58E-02 | 48 |
| Toxoplasmosis | 0.29 | 1.89E-02 | 64 |
| Thyroid_cancer | 0.48 | 2.44E-02 | 22 |
| ErbB_signaling_pathway | 0.27 | 2.88E-02 | 67 |
| Insulin_signaling_pathway | 0.23 | 2.95E-02 | 91 |
| NOD-like_receptor_signaling_pathway | 0.40 | 3.36E-02 | 28 |
| Jak-STAT_signaling_pathway | 0.28 | 3.41E-02 | 56 |
| Nucleotide_excision_repair | 0.36 | 3.53E-02 | 34 |
| VEGF_signaling_pathway | 0.30 | 4.01E-02 | 46 |
| Non-small_cell_lung_cancer | 0.31 | 4.76E-02 | 41 |
| Acute_myeloid_leukemia | 0.30 | 4.84E-02 | 44 |
| Adipocytokine_signaling_pathway | 0.29 | 5.64E-02 | 44 |
| Lysosome | 0.20 | 5.85E-02 | 93 |
| Renal_cell_carcinoma | 0.24 | 6.02E-02 | 62 |
| Neurotrophin_signaling_pathway | 0.19 | 6.15E-02 | 98 |
| Pancreatic_cancer | 0.25 | 6.60E-02 | 56 |
| Purine_metabolism | 0.17 | 7.42E-02 | 114 |
| Sphingolipid_metabolism | 0.35 | 7.44E-02 | 27 |
| Epithelial_cell_signaling_in_Helicobacter_pylori_infection | 0.25 | 7.71E-02 | 50 |
| Endocytosis | 0.15 | 7.96E-02 | 139 |
| Prion_diseases | -0.40 | 8.62E-02 | 19 |
| Cell_adhesion_molecules_(CAMs) | 0.24 | 8.81E-02 | 52 |
| Colorectal_cancer | 0.24 | 9.17E-02 | 50 |
| Progesterone-mediated_oocyte_maturation | 0.21 | 9.22E-02 | 63 |
| Natural_killer_cell_mediated_cytotoxicity | 0.24 | 9.33E-02 | 51 |
| Glyoxylate_and_dicarboxylate_metabolism | 0.45 | 9.39E-02 | 15 |
| Ribosome | 0.38 | 9.44E-02 | 20 |
| Regulation_of_autophagy | 0.45 | 9.52E-02 | 15 |
| Leishmaniasis | 0.32 | 1.00E-01 | 28 |
| Glycosaminoglycan_degradation | 0.49 | 1.05E-01 | 12 |
| Toll-like_receptor_signaling_pathway | 0.24 | 1.06E-01 | 48 |
| Regulation_of_actin_cytoskeleton | 0.13 | 1.24E-01 | 132 |
| Tryptophan_metabolism | 0.36 | 1.28E-01 | 19 |
| Hypertrophic_cardiomyopathy_(HCM) | 0.23 | 1.30E-01 | 46 |
| Mineral_absorption | -0.33 | 1.31E-01 | 22 |
| GnRH_signaling_pathway | 0.19 | 1.34E-01 | 62 |
| Arrhythmogenic_right_ventricular_cardiomyopathy_(ARVC) | 0.23 | 1.45E-01 | 42 |
| Viral_myocarditis | -0.32 | 1.50E-01 | 22 |
| Glycerophospholipid_metabolism | 0.19 | 1.51E-01 | 56 |
| Chemokine_signaling_pathway | 0.14 | 1.56E-01 | 99 |
| Bacterial_invasion_of_epithelial_cells | 0.20 | 1.60E-01 | 52 |
| Protein_processing_in_endoplasmic_reticulum | 0.13 | 1.64E-01 | 124 |
| Base_excision_repair | -0.31 | 1.74E-01 | 21 |
| Fatty_acid_metabolism | 0.27 | 1.79E-01 | 26 |
| Long-term_potentiation | 0.19 | 1.79E-01 | 52 |
| Pyruvate_metabolism | 0.25 | 1.87E-01 | 30 |
| SNARE_interactions_in_vesicular_transport | 0.26 | 1.89E-01 | 28 |
| Osteoclast_differentiation | 0.16 | 1.94E-01 | 65 |
| Neuroactive_ligand-receptor_interaction | 0.13 | 1.96E-01 | 97 |
| Amino_sugar_and_nucleotide_sugar_metabolism | 0.21 | 2.02E-01 | 37 |
| Hepatitis_C | 0.14 | 2.14E-01 | 82 |
| Shigellosis | 0.19 | 2.17E-01 | 45 |
| Basal_transcription_factors | 0.23 | 2.25E-01 | 29 |
| Phosphatidylinositol_signaling_system | 0.16 | 2.32E-01 | 60 |
| Lysine_degradation | 0.21 | 2.34E-01 | 33 |
| Drug_metabolism_-_other_enzymes | 0.37 | 2.35E-01 | 12 |
| Glycolysis_/_Gluconeogenesis | 0.23 | 2.38E-01 | 28 |
| Butanoate_metabolism | -0.32 | 2.44E-01 | 15 |
| Nicotinate_and_nicotinamide_metabolism | 0.36 | 2.44E-01 | 12 |
| Melanogenesis | 0.16 | 2.48E-01 | 54 |
| Mismatch_repair | 0.28 | 2.53E-01 | 18 |
| Huntington_disease | 0.10 | 2.60E-01 | 123 |
| Small_cell_lung_cancer | 0.15 | 2.60E-01 | 55 |
| Alanine,_aspartate_and_glutamate_metabolism | 0.25 | 2.62E-01 | 22 |
| Arginine_and_proline_metabolism | 0.20 | 2.97E-01 | 29 |
| Axon_guidance | 0.11 | 2.98E-01 | 90 |
| Endometrial_cancer | 0.17 | 3.02E-01 | 40 |
| Chagas_disease_(American_trypanosomiasis) | 0.13 | 3.07E-01 | 60 |
| Mucin_type_O-Glycan_biosynthesis | 0.26 | 3.11E-01 | 17 |
| Cytosolic_DNA-sensing_pathway | 0.19 | 3.14E-01 | 29 |
| TGF-beta_signaling_pathway | -0.14 | 3.18E-01 | 50 |
| Fc_gamma_R-mediated_phagocytosis | 0.13 | 3.21E-01 | 63 |
| Valine,_leucine_and_isoleucine_degradation | 0.17 | 3.25E-01 | 36 |
| Cardiac_muscle_contraction | 0.15 | 3.26E-01 | 43 |
| Hedgehog_signaling_pathway | -0.20 | 3.28E-01 | 25 |
| Cysteine_and_methionine_metabolism | -0.22 | 3.35E-01 | 21 |
| Circadian_rhythm_-_mammal | 0.24 | 3.37E-01 | 18 |
| Alzheimer_disease | 0.09 | 3.39E-01 | 110 |
| Aldosterone-regulated_sodium_reabsorption | 0.19 | 3.49E-01 | 27 |
| N-Glycan_biosynthesis | 0.17 | 3.53E-01 | 32 |
| mTOR_signaling_pathway | 0.14 | 3.68E-01 | 42 |
| Dilated_cardiomyopathy | 0.12 | 3.90E-01 | 51 |
| Oocyte_meiosis | -0.10 | 3.91E-01 | 74 |
| Focal_adhesion | 0.07 | 3.93E-01 | 133 |
| Basal_cell_carcinoma | 0.18 | 4.02E-01 | 23 |
| Pathogenic_Escherichia_coli_infection | -0.16 | 4.03E-01 | 31 |
| Porphyrin_and_chlorophyll_metabolism | 0.21 | 4.10E-01 | 17 |
| Ubiquitin_mediated_proteolysis | 0.08 | 4.27E-01 | 101 |
| Protein_export | -0.21 | 4.32E-01 | 16 |
| Other_glycan_degradation | 0.26 | 4.37E-01 | 11 |
| Tyrosine_metabolism | 0.22 | 4.50E-01 | 14 |
| Pentose_phosphate_pathway | 0.18 | 4.64E-01 | 19 |
| Glycosaminoglycan_biosynthesis_-_chondroitin_sulfate | -0.22 | 5.00E-01 | 12 |
| Leukocyte_transendothelial_migration | 0.09 | 5.05E-01 | 63 |
| Amyotrophic_lateral_sclerosis_(ALS) | 0.11 | 5.17E-01 | 35 |
| Protein_digestion_and_absorption | -0.12 | 5.26E-01 | 31 |
| Inositol_phosphate_metabolism | 0.10 | 5.26E-01 | 40 |
| Pyrimidine_metabolism | 0.08 | 5.35E-01 | 67 |
| Terpenoid_backbone_biosynthesis | 0.21 | 5.36E-01 | 11 |
| ECM-receptor_interaction | 0.10 | 5.45E-01 | 40 |
| Gap_junction | 0.08 | 5.49E-01 | 60 |
| Phagosome | -0.07 | 5.50E-01 | 66 |
| Selenocompound_metabolism | -0.19 | 5.51E-01 | 12 |
| RNA_polymerase | 0.13 | 5.52E-01 | 24 |
| RIG-I-like_receptor_signaling_pathway | 0.10 | 5.55E-01 | 34 |
| Glycosphingolipid_biosynthesis_-_lacto_and_neolacto_series | -0.17 | 5.63E-01 | 14 |
| Proteasome | 0.10 | 5.70E-01 | 35 |
| Taste_transduction | -0.17 | 5.81E-01 | 13 |
| Glycosaminoglycan_biosynthesis_-_heparan_sulfate | 0.16 | 5.83E-01 | 14 |
| Ribosome_biogenesis_in_eukaryotes | -0.07 | 5.91E-01 | 54 |
| Drug_metabolism_-_cytochrome_P450 | 0.17 | 5.95E-01 | 12 |
| Malaria | 0.14 | 5.96E-01 | 16 |
| p53_signaling_pathway | -0.09 | 6.09E-01 | 38 |
| Amoebiasis | -0.07 | 6.14E-01 | 50 |
| Bile_secretion | -0.09 | 6.19E-01 | 36 |
| Adherens_junction | 0.07 | 6.26E-01 | 53 |
| Carbohydrate_digestion_and_absorption | 0.11 | 6.35E-01 | 22 |
| Histidine_metabolism | -0.13 | 6.39E-01 | 15 |
| Long-term_depression | 0.07 | 6.45E-01 | 48 |
| Glycerolipid_metabolism | -0.08 | 6.58E-01 | 33 |
| Spliceosome | 0.05 | 6.64E-01 | 90 |
| Olfactory_transduction | -0.13 | 6.72E-01 | 13 |
| Hematopoietic_cell_lineage | -0.10 | 6.85E-01 | 18 |
| beta-Alanine_metabolism | -0.11 | 6.92E-01 | 15 |
| Other_types_of_O-glycan_biosynthesis | -0.09 | 6.97E-01 | 21 |
| ABC_transporters | 0.08 | 7.10E-01 | 22 |
| Pentose_and_glucuronate_interconversions | -0.12 | 7.15E-01 | 11 |
| Cytokine-cytokine_receptor_interaction | -0.04 | 7.30E-01 | 64 |
| Gastric_acid_secretion | -0.05 | 7.47E-01 | 46 |
| Rheumatoid_arthritis | 0.06 | 7.47E-01 | 32 |
| Complement_and_coagulation_cascades | 0.08 | 7.53E-01 | 19 |
| Collecting_duct_acid_secretion | 0.09 | 7.58E-01 | 15 |
| Wnt_signaling_pathway | 0.03 | 7.61E-01 | 90 |
| Systemic_lupus_erythematosus | 0.07 | 7.68E-01 | 21 |
| Vasopressin-regulated_water_reabsorption | -0.06 | 7.72E-01 | 29 |
| Vascular_smooth_muscle_contraction | -0.04 | 7.75E-01 | 67 |
| Fructose_and_mannose_metabolism | 0.06 | 7.85E-01 | 25 |
| Type_II_diabetes_mellitus | -0.05 | 7.87E-01 | 27 |
| Homologous_recombination | 0.07 | 7.92E-01 | 16 |
| Biosynthesis_of_unsaturated_fatty_acids | 0.07 | 7.96E-01 | 16 |
| Ether_lipid_metabolism | 0.06 | 8.09E-01 | 21 |
| Oxidative_phosphorylation | -0.03 | 8.14E-01 | 82 |
| Propanoate_metabolism | 0.05 | 8.15E-01 | 25 |
| Peroxisome | 0.03 | 8.23E-01 | 59 |
| RNA_transport | -0.02 | 8.23E-01 | 103 |
| mRNA_surveillance_pathway | -0.03 | 8.24E-01 | 55 |
| Proximal_tubule_bicarbonate_reclamation | -0.07 | 8.25E-01 | 13 |
| RNA_degradation | -0.03 | 8.49E-01 | 52 |
| Glycine,_serine_and_threonine_metabolism | -0.05 | 8.50E-01 | 18 |
| Dorso-ventral_axis_formation | -0.05 | 8.56E-01 | 16 |
| Bladder_cancer | -0.03 | 8.76E-01 | 30 |
| Aminoacyl-tRNA_biosynthesis | 0.03 | 8.80E-01 | 35 |
| Starch_and_sucrose_metabolism | 0.04 | 8.83E-01 | 17 |
| Arachidonic_acid_metabolism | -0.04 | 8.95E-01 | 16 |
| Fat_digestion_and_absorption | -0.04 | 8.95E-01 | 13 |
| Antigen_processing_and_presentation | 0.03 | 9.02E-01 | 24 |
| Salivary_secretion | -0.02 | 9.16E-01 | 41 |
| Galactose_metabolism | 0.02 | 9.27E-01 | 18 |
| Tight_junction | 0.01 | 9.28E-01 | 82 |
| Vibrio_cholerae_infection | -0.01 | 9.47E-01 | 37 |
| Citrate_cycle_(TCA_cycle) | -0.01 | 9.59E-01 | 27 |
| Parkinson_disease | -0.01 | 9.62E-01 | 78 |
| PPAR_signaling_pathway | -0.01 | 9.62E-01 | 31 |
| Glutathione_metabolism | -0.01 | 9.64E-01 | 27 |
| Apoptosis | 0.01 | 9.67E-01 | 53 |
| DNA_replication | 0.00 | 9.81E-01 | 27 |
| Cell_cycle | 0.00 | 9.89E-01 | 81 |
| Pancreatic_secretion | 0.00 | 9.95E-01 | 46 |
| Glycosylphosphatidylinositol(GPI)-anchor_biosynthesis | 0.00 | 9.99E-01 | 20 |
| African_trypanosomiasis | 0.00 | 1.00E+00 | 0 |
| Allograft_rejection | 0.00 | 1.00E+00 | 0 |
| alpha-Linolenic_acid_metabolism | 0.00 | 1.00E+00 | 0 |
| Ascorbate_and_aldarate_metabolism | 0.00 | 1.00E+00 | 0 |
| Asthma | 0.00 | 1.00E+00 | 0 |
| Autoimmune_thyroid_disease | 0.00 | 1.00E+00 | 0 |
| Biotin_metabolism | 0.00 | 1.00E+00 | 0 |
| Butirosin_and_neomycin_biosynthesis | 0.00 | 1.00E+00 | 0 |
| Caffeine_metabolism | 0.00 | 1.00E+00 | 0 |
| Cyanoamino_acid_metabolism | 0.00 | 1.00E+00 | 0 |
| D-Arginine_and_D-ornithine_metabolism | 0.00 | 1.00E+00 | 0 |
| D-Glutamine_and_D-glutamate_metabolism | 0.00 | 1.00E+00 | 0 |
| Fatty_acid_biosynthesis | 0.00 | 1.00E+00 | 0 |
| Fatty_acid_elongation_in_mitochondria | 0.00 | 1.00E+00 | 0 |
| Folate_biosynthesis | 0.00 | 1.00E+00 | 0 |
| Glycosaminoglycan_biosynthesis_-_keratan_sulfate | 0.00 | 1.00E+00 | 0 |
| Glycosphingolipid_biosynthesis_-_ganglio_series | 0.00 | 1.00E+00 | 0 |
| Glycosphingolipid_biosynthesis_-_globo_series | 0.00 | 1.00E+00 | 0 |
| Graft-versus-host_disease | 0.00 | 1.00E+00 | 0 |
| Intestinal_immune_network_for_IgA_production | 0.00 | 1.00E+00 | 0 |
| Linoleic_acid_metabolism | 0.00 | 1.00E+00 | 0 |
| Lipoic_acid_metabolism | 0.00 | 1.00E+00 | 0 |
| Lysine_biosynthesis | 0.00 | 1.00E+00 | 0 |
| Maturity_onset_diabetes_of_the_young | 0.00 | 1.00E+00 | 0 |
| Metabolism_of_xenobiotics_by_cytochrome_P450 | 0.00 | 1.00E+00 | 0 |
| Nitrogen_metabolism | 0.00 | 1.00E+00 | 0 |
| Non-homologous_end-joining | 0.00 | 1.00E+00 | 0 |
| One_carbon_pool_by_folate | 0.00 | 1.00E+00 | 0 |
| Pantothenate_and_CoA_biosynthesis | 0.00 | 1.00E+00 | 0 |
| Phenylalanine_metabolism | 0.00 | 1.00E+00 | 0 |
| Phenylalanine,_tyrosine_and_tryptophan_biosynthesis | 0.00 | 1.00E+00 | 0 |
| Phototransduction | 0.00 | 1.00E+00 | 0 |
| Primary_bile_acid_biosynthesis | 0.00 | 1.00E+00 | 0 |
| Primary_immunodeficiency | 0.00 | 1.00E+00 | 0 |
| Renin-angiotensin_system | 0.00 | 1.00E+00 | 0 |
| Retinol_metabolism | 0.00 | 1.00E+00 | 0 |
| Riboflavin_metabolism | 0.00 | 1.00E+00 | 0 |
| Staphylococcus_aureus_infection | 0.00 | 1.00E+00 | 0 |
| Steroid_biosynthesis | 0.00 | 1.00E+00 | 0 |
| Steroid_hormone_biosynthesis | 0.00 | 1.00E+00 | 0 |
| Sulfur_metabolism | 0.00 | 1.00E+00 | 0 |
| Sulfur_relay_system | 0.00 | 1.00E+00 | 0 |
| Synthesis_and_degradation_of_ketone_bodies | 0.00 | 1.00E+00 | 0 |
| Taurine_and_hypotaurine_metabolism | 0.00 | 1.00E+00 | 0 |
| Thiamine_metabolism | 0.00 | 1.00E+00 | 0 |
| Type_I_diabetes_mellitus | 0.00 | 1.00E+00 | 0 |
| Ubiquinone_and_other_terpenoid-quinone_biosynthesis | 0.00 | 1.00E+00 | 0 |
| Valine,_leucine_and_isoleucine_biosynthesis | 0.00 | 1.00E+00 | 0 |
| Vitamin_B6_metabolism | 0.00 | 1.00E+00 | 0 |
| Vitamin_digestion_and_absorption | 0.00 | 1.00E+00 | 0 |
